# Epidemiological and clinical characteristics of monkeypox cases in Brazil in 2022: a cross-sectional study

**DOI:** 10.1590/S2237-96222022000300036

**Published:** 2022-12-19

**Authors:** Ana Roberta Pati Pascom, Isabella Nepomuceno de Souza, Amanda Krummenauer, Magda Machado Saraiva Duarte, Janaina Sallas, Daniela Buosi Rohlfs, Gerson Mendes Pereira, Arnaldo Correia de Medeiros, Angélica Espinosa Miranda

**Affiliations:** 1 Ministério da Saúde, Secretaria de Vigilância em Saúde, Brasília, DF, Brazil

**Keywords:** Monkeypox Virus, Monkeypox, Outbreaks, Disease Outbreaks, Epidemiology, Brazil

## Abstract

**Objective:**

to describe epidemiological and clinical characteristics of monkeypox (MPX) in Brazil, from the identification of the first case, on June 7, 2022, to Epidemiological Week (EW) 39, ending on October 1, 2022.

**Methods:**

this was a descriptive study of cases notified to the Ministry of Health; trends were analyzed based on the number of confirmed and probable cases per EW; the cases were also described according to demographic and clinical variables.

**Results:**

out of 31,513 notifications, 23.8% were confirmed; 91.8% were male; 70.6% were cis men; and median age was 32 years. Fever (58.0%), adenomegaly (42.4%), headache (39.9%) and rash (37.0%) were the most frequent symptoms; 27.5% reported being immunosuppressed, 34.6% were living with HIV and 10.5% had a sexually transmitted infection; three deaths were recorded.

**Conclusion:**

the MPX case profile was similar to that of other countries; surveillance actions must be strengthened to control the outbreak.

**Study contributions**

**Main results**

High monkeypox (MPX) prevalence among cis men with a median age of 32 years. Fever was the most frequent symptom, followed by adenomegaly, headache and rash. High proportions of MPX in people living with HIV and other STIs, as in other countries.

**Implications for services**

Disseminating data about MPX contributes to consolidating the scientific literature in Brazil. The continuous and systematic process of analyzing events like this assists with planning and implementing health surveillance measures.

**Perspectives**

In order to complement the findings of this study, the progression of the disease in Brazil needs to be monitored and new studies are needed in order to enhance health surveillance work.

## Introduction

The first case of monkeypox (MPX) in humans was registered in the Democratic Republic of Congo in 1970. Since then, the disease has become endemic in some African countries and sporadic outbreaks outside Africa have been reported in recent years.^1^ ^-^ ^3^ In early 2022, a systematic review conducted by Bunge et al. highlighted the risk of the worldwide spread of the disease and warned of the possibility of MPX becoming a pandemic.^4^ On May 7, 2022, the United Kingdom reported the occurrence of a confirmed MPX case in that country and following that its spread to other non-endemic countries was reported.^3^

In the face of MPX spreading to 72 countries and with almost 15,000 confirmed cases, on July 23, 2022, the World Health Organization (WHO) declared the disease to be a Public Health Emergency of International Concern (PHEIC), raised the level of attention paid to the disease and recommended the expansion of surveillance capacity and public health measures to contain its transmission in countries worldwide.^5^

In Brazil, on May 23, 2022, the Health Ministry’s Health Surveillance Secretariat (*Secretaria de Vigilância em Saúde do Ministério da Saúde* - SVS/MS) mobilized its Situation Room in order to organize and prepare the Brazilian National Health System (*Sistema Único de Saúde* - SUS) to face the disease. With the advance of MPX in the country, on July 29, 2022, the SVS/MS mobilized the National Public Health Emergency Operations Center (*Centro de Operações de Emergência em Saúde Pública Nacional* - COE-MPX), which has acted to organize and coordinate SUS actions in response to the disease, strengthen surveillance and adopt prevention and control measures to contain the emergency at the federal, state and municipal management levels of the Health System.^6^

On June 7, 2022, the first MPX case was confirmed in the state of São Paulo, and routine surveillance was put in place on July 12. Since then, notification/investigation of suspected MPX cases has become mandatory and immediate (within 24 hours) throughout the national territory, by health professionals working in public and private services, as determined by Law No. 6,259, dated October 30, 1975.^7^ On August 31, 2022, MPX was included on the National List of Compulsorily Notifiable diseases and public health problems.^8^

Dissemination of data on MPX contributes to the consolidation of scientific literature in Brazil. The continuous and systematic process of analyzing similar events can assist with planning and implementation of health surveillance measures.

The objective of this study was to describe the origin and the epidemiological and clinical characteristics of confirmed and probable MPX cases in Brazil.

## Methods

This was a descriptive cross-sectional study of MPX cases notified to the Ministry of Health since the identification of the first case, on June 7, epidemiological week 23, until the end of the 39^th^ epidemiological week (EW), on October 1, 2022, and recorded on the official notification systems by October 6, 2022. Although the state of São Paulo has its own notification form and system, the information about cases reported in that state was made compatible and consolidated with the information for the rest of the country held on the national system and then included in this analysis. In situations in which compatibility was not possible, we have presented national system information, with the exception of São Paulo.

All suspected cases that were classified as confirmed or probable, according to the case definition criteria established by the Ministry of Health on August 5, 2022, were included in the present study.^9^ According to that definition, suspected cases were considered to be individuals, of any age, who presented sudden onset of mucosal lesions and/or acute skin rash suggestive of MPX and/or proctitis and/or penile edema, whether or not associated with other signs and symptoms. Among the suspected cases, those with positive or detectable MPX virus laboratory test results using molecular diagnosis through real-time polymerase chain reaction (PCR) and/or sequencing were considered to be confirmed; cases with negative or non-detectable results were discarded. 

Suspected cases of MPX were classified as being probable cases when laboratory tests were not performed or had inconclusive results, when diagnosis of MPX could not be ruled out only by clinical-laboratorial confirmation of diagnosis of another health condition, and who reported exposure or close contact with a confirmed case of MPX in the 21 days prior to the onset of signs and symptoms.^9^

Cases were described according to the following sociodemographic characteristics: national macro-region of residence (North; Northeast; Southeast; South; Midwest), sex at birth (male; female), gender (trans women; cis women; transvestite; trans men; cis men; non-binary; other); age group (in completed years: up to 1; 1-4; 5-9; 10-14; 15-17; 18-29; 30-39; 40-49; 50-59; 60 or over); and race/skin color [White; Black (mixed race and Black); Asian; Indigenous]. In the case of records with male sex at birth, we described: (i) sexual orientation (heterosexual; homosexual; bisexual; pansexual; other), except for case notifications made in the state of São Paulo which did not contain this information on the notification form; and (ii) sexual behavior, defining as gay men and other men who have sex with men (MSM) males who reported having sex only with men, or with men and with women, respectively.

Cases were also analyzed according to: (i) presence of signs and symptoms (fever; rash; headache; adenomegaly; muscle pain; asthenia; backache; genital lesion; sore throat; sweating or shivering; localized lymphadenopathy; nausea; arthralgia; oral lesion; proctitis; penile edema; cough; mucosal lesion; photosensitivity; hemorrhage; generalized lymphadenopathy; diarrhea; conjunctivitis; other); (ii) site on the body where lesions and rash appeared (genital region; torso; upper limbs; face; lower limbs; anal region; palm of the hand; oral region; sole of the foot; other sites); (iii) presence of immunosuppression described on the notification form (yes, due to a prior disease; yes, due to medication; yes, cause unknown; not immunosuppressed); (iv) self-reported human immunodeficiency virus (HIV) infection (yes; no); and (v) self-reported active sexually transmitted infection (STI) (syphilis; genital herpes; chlamydia; gonorrhea; genital warts; other STIs; did not have an active STI). 

With the exception of the state of São Paulo, which did not have this information on its notification forms, we described cases according to: (i) need for hospitalization (yes, due to clinical reasons related to MPX; yes, due to need to isolate the case); and (ii) hospitalization in intensive care units, ICU (yes; no). Finally, we presented the progression of confirmed and probable cases according to cure, death due to MPX or death due to another cause; and, for cases notified in São Paulo, whether the individual was still under treatment.

The MPX incidence rates, according to sex at birth and Federative Unit of residence, were calculated by dividing notified confirmed and probable cases (numerator) by the respective estimated populations for the period^10^ (denominator), multiplied by 100,000. The incidence ratio according to sex at birth was also calculated.

The MPX trends were presented according to the number of confirmed and probable cases notified per EW, according to the date of notification. In addition, we calculated the moving averages of the number of daily cases notified, per EW of notification. 

The cases were described according to absolute and relative frequency. We also calculated the median and the interquartile range (IQR) for the “age” variable. We used the Statistical Package for the Social Sciences (IBM SPSS for Windows), release 25.0, in all the analyses. 

All the analyses presented in this paper were performed using secondary data notified on the official MPX surveillance systems in Brazil, but without considering any variables that could allow the people in question to be identified. A unique code was assigned by the Ministry of Health to all cases before this analysis was carried out, in order to guarantee secrecy and confidentiality of each notified case. The data are published in a different analysis format in the MPX information bulletins.^9^ Consequently, the study project was exempted from submission to and appraisal by a Research Ethics Committee, but was in accordance with National Health Council Resolution No. 466, dated December 12, 2012.

## Results

In Brazil, as at EW 39, which ended on October 1, 2022, 33,513 notified MPX cases had been recorded, representing an increase of 7% in the number of cumulative notifications as at EW 38 (31,284). Of this total, 58.6% of notifications were discarded (19,649), 23.8% (7,992) were confirmed, and 0.5% (175) were classified as probable. 

**Figure 1:**
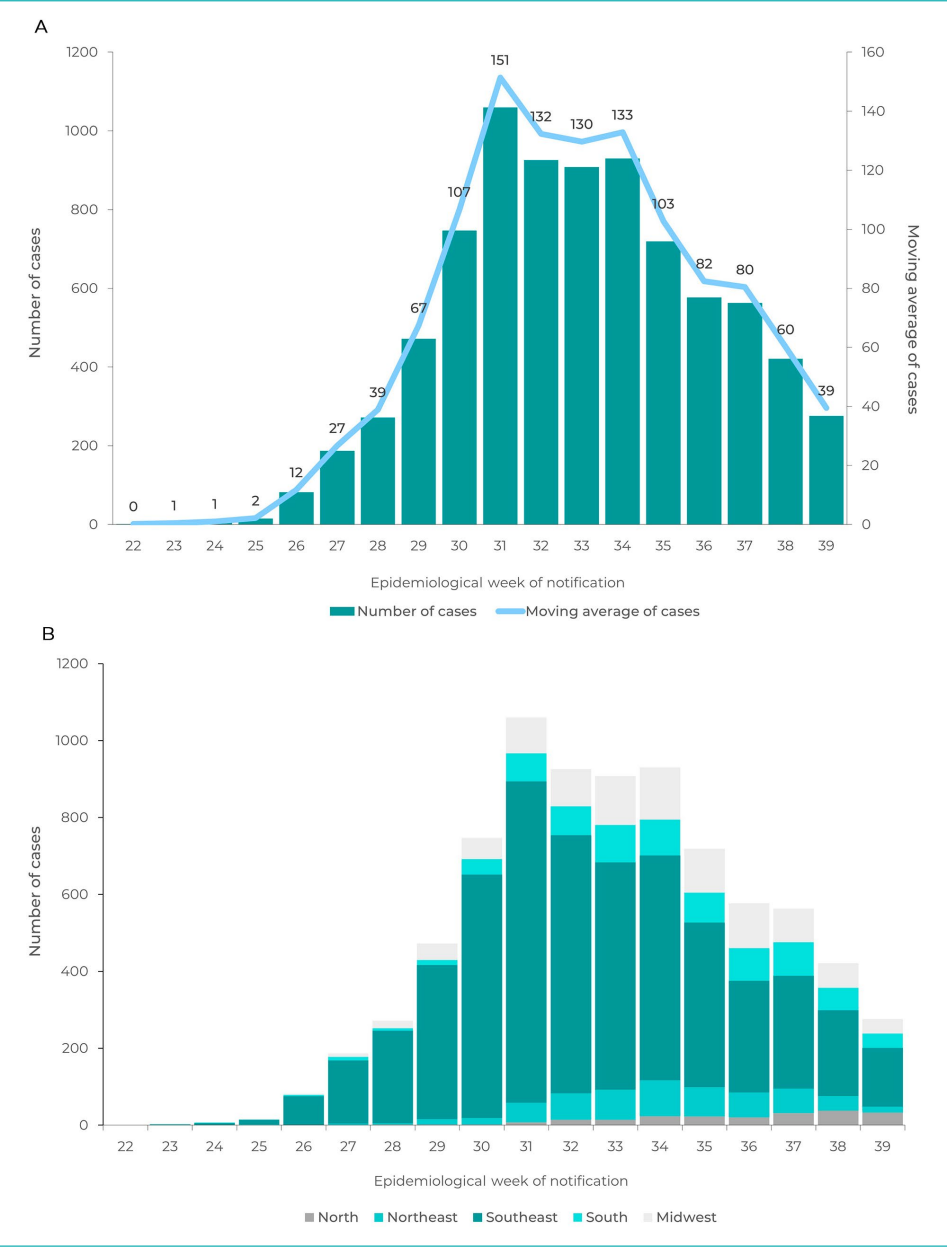
Number of confirmed and probable monkeypox cases and moving averages (A), and case distribution per region of residence (B), by epidemiological week of case notification, Brazil, June 7, 2022 (EW 23) - October 1, 2022 (EW 39)

Figure 1A shows the distribution of 8,167 confirmed and probable cases, according to the EW of notification and their respective moving averages. An increase in the number of cases was found up until EW 30, when it reached around 150 confirmed and probable cases per day. After this period, the daily number of cases remained stable at around 130 cases per day between EW 31 and EW 33. After this a falling trend was found. In EW 39, an average of 39 confirmed and probable cases per day were notified. 

Even with the slowdown in the growth of the number of cases in the last EWs (with effect from EW 35), MPX spread throughout the country (Figure 1B). Up until EW 28, the Southeast region concentrated more than 85% of confirmed and probable cases, and the Midwest region, 7%. As at EW 38, 53% of confirmed cases were in the Southeast region, 15% in the Midwest region and 14% in the Southern region. It is also noteworthy that as at EW 39, 497 municipalities had reported at least one confirmed or probable case.

Table 1 shows that the MPX incidence rate in Brazil was 3.8 cases per 100,000 inhabitants as at SE 39. Regarding sex, the incidence of infection was 12 times higher in males (7.2/100,000) when compared to females (0.6/100,000). Moreover, the highest concentration of confirmed and probable cases was found in the Southeast (n = 5,607; 68.7%) and Midwest (n = 998; 12.2%) regions; consequently, the highest incidence rates were found in these two regions (6.3 and 6.0 cases per 100,000 inhabitants, respectively).

**Table 1:** Number of confirmed and probable monkeypox cases and incidence rate, according to sex at birth, by Federative Unit, Brazil, June 7, 2022 (EW 23) - October 1, 2022 (EW 39)

Region/Federative Unit	Number of cases			Incidence rate (per 100,000 inhab.)		
	Male	Female	Total^a^	Male	Female	Total^a^
Brazil	7,494	653	8,167	7.2	0.6	3.8
North	205	8	213	2.2	0.1	1.1
Rondônia	6	1	7	0.7	0.1	0.4
Acre	1	-	1	0.2	0.0	0.1
Amazonas	143	4	147	6.7	0.2	3.4
Roraima	4	-	4	1.2	0.0	0.6
Pará	40	1	41	0.9	0.0	0.5
Amapá	2	-	2	0.5	0.0	0.2
Tocantins	9	2	11	1.1	0.3	0.7
Northeast	489	100	589	1.8	0.3	1.0
Maranhão	19	-	19	0.5	0.0	0.3
Piauí	14	1	15	0.9	0.1	0.5
Ceará	112	18	130	2.5	0.4	1.4
Rio Grande do Norte	74	19	93	4.3	1.0	2.6
Paraíba	26	3	29	1.3	0.1	0.7
Pernambuco	127	35	162	2.7	0.7	1.7
Alagoas	11	3	14	0.7	0.2	0.4
Sergipe	10	4	14	0.9	0.3	0.6
Bahia	96	17	113	1.3	0.2	0.8
Southeast	5,160	429	5,607	11.8	0.9	6.3
Minas Gerais	519	15	535	4.9	0.1	2.5
Espírito Santo	66	10	78	3.3	0.5	1.9
Rio de Janeiro	1,050	98	1,148	12.6	1.1	6.6
São Paulo	3,525	306	3,846	15.5	1.3	8.2
South	695	65	760	4.7	0.4	2.5
Paraná	219	15	234	3.9	0.3	2.0
Santa Catarina	279	21	300	7.7	0.6	4.1
Rio Grande do Sul	197	29	226	3.5	0.5	2.0
Midwest	945	51	998	11.4	0.6	6.0
Mato Grosso do Sul	113	13	126	8.0	0.9	4.4
Mato Grosso	80	4	85	4.4	0.2	2.4
Goiás	490	24	515	13.7	0.7	7.1
Federal District	262	10	272	17.6	0.6	8.8

a) Total cases refer to notified cases, including the 20 cases with no information on sex at birth (15 in São Paulo, 2 in Espírito Santo, 1 in Minas Gerais, 1 in Goiás and 1 in the Federal District).

Most confirmed and probable cases occurred among individuals living in the states of São Paulo (n = 3,846; 47.1%), Rio de Janeiro (n = 1,148; 14.1%), Minas Gerais (n = 535; 6.6%), and Goiás (n = 515; 6.3%), totaling almost a third of the national cases. These were also the four federative units among those with the highest incidence rates: 8.8 cases/100,000 in the Federal District; 8.2 cases/100,000 in São Paulo; 7.1 cases/100,000 in Goiás; and 6.6 cases/100,000 in Rio de Janeiro.

As for the demographic characteristics of confirmed and probable cases, 91.8% (7,494) were born male. 70.6% (5,766) of the notified cases were self-reported cis men, while this information was not available for 19.2% (1,564). The median age of the cases analyzed was 32 years (IQR 27-38 years), of which 40.6% (3,314) were individuals aged 30 to 39 years and 34.3% (2,805) were between 18 and 29 years old. Ten cases were reported in children under 1 year of age and 218 (2.7%) in children aged 1 to 9 years; 43.7% (3,572) of the cases reported being White, 40.8% (3,332) Black, and 13 self-reported being of Indigenous race/skin color (Table 2).

**Table 2:** Number and proportion of confirmed and probable monkeypox cases (n = 8,167) according to demographic characteristics, sexual orientation and sexual behavior, Brazil, June 7, 2022 (EW 23) - October 1, 2022 (EW 39)

Characteristics	n	%
Total	8,167	100.0
**Sex at birth**		
Male	7,494	91.8
Female	653	8.0
Not informed	20	0.2
**Gender**		
Trans women	21	0.3
Cis women	521	6.3
Transvestite	6	0.1
Trans men	115	1.4
Cis men	5,766	70.6
Non-binary	70	0.9
Other	104	1.3
Not informed	1,564	19.1
**Age group (in completed years)**		
< 1	10	0.1
1-4	80	1.0
5-9	67	0.8
10-14	71	0.9
15-17	69	0.8
18-29	2,805	34.3
30-39	3,314	40.6
40-49	1,349	16.5
50-59	314	3.9
≥ 60	84	1.0
Not informed	4	0.1
**Race/skin color**		
White	3,572	43.7
Black	3,332	40.8
Asian	87	1.1
Indigenous	13	0.2
Not informed	1,163	14.2
**Region of residence**		
North	213	2.6
Northeast	589	7.2
Southeast	5,607	68.7
South	760	9.3
Midwest	998	12.2
Not informed	-	0
**Sexual orientation^a^**		
Heterosexual	534	7.1
Homosexual	2,566	34.2
Bisexual	379	5.1
Pansexual	72	1.0
Other	360	4.8
Not informed^b^	3,583	47.8
**Gay men and other MSM^a,c^**		
Yes	4502	60.1
No	687	9.2
Not informed	2,305	30.8

a) Refers only to the 7,494 cases whose sex was male at birth; b) Includes 3,523 notifications of individual whose sex was male at birth living in São Paulo with no information on sexual orientation; c) Gay men and other MSM (men who have sex with men) were defined as those whose sex at birth was male and who reported only having sex with men or having sex with men and women.

Male cases that did not inform their sexual orientation accounted for 47.8% (3,583), while 34.2% (2,566) self-reported being homosexual. Of the cases with no record of sexual orientation, 3,523 were reported in São Paulo, since this information was not included on that state’s notification form. Of the cases notified in the rest of the country, 64.6% (2,566) self-reported being homosexual, 9.5% (379) bisexual, 1.8% (72) pansexual, and 9.1% (360) reported having another sexual orientation. With regard to the signs and symptoms reported among MPX cases, the most frequent were fever (n = 4,709; 57.7%), rash (n = 3,498; 42.3%), headache (n = 3,280; 40.2%), and adenomegaly (n = 3,255; 39.9%). Genital lesions were reported by 18.9% (1,542), oral lesions by 4% (359) and 3.2% (265) reported mucosal lesions. Most cases (n = 3,913; 47.9%) stated that lesions and/or rash initially appeared in the genital region, followed by the torso (n = 2,893; 35.4%) and upper limbs (n = 2,832; 34.7%). Almost one fifth (n = 1,585) of the cases reported lesions and/or rash in the anal region (Table 3).

**Table 3:** Number and proportion of confirmed and probable monkeypox cases (n = 8,167) according to presence of signs and symptoms and site on the body where lesions or rash appeared, Brazil, June 7, 2022 (EW 23) - October 1, 2022 (EW 39)

Characteristics	n	%
Total	8,167	100.0
**Signs and symptoms**		
At least one sign	7,399	90.6
Fever	4,709	57.7
Rash	3,498	42.8
Headache	3,280	40.2
Adenomegaly	3,255	39.9
Muscle pain	2,919	35.7
Asthenia	2,679	32.8
Backache	1,586	19.4
Genital lesion	1,542	18.9
Sore throat	1,108	13.6
Sweating/shivering	1,070	13.1
Localized lymphadenopathy	828	10.1
Nausea	470	5.8
Arthralgia	398	4.9
Oral lesion	359	4.4
Proctitis	338	4.1
Penile edema	332	4.1
Cough	300	3.7
Mucosal lesion	265	3.2
Photosensitivity	167	2.0
Hemorrhage	99	1.2
Generalized lymphadenopathy	84	1.0
Diarrhea	84	1.0
Conjunctivitis	81	1.0
Other signs and symptoms	755	9.2
**Site of lesion or rash**		
Genital region	3,913	47.9
Torso	2,893	35.4
Upper limbs	2,832	34.7
Face	2,260	27.7
Lower limbs	2,092	25.6
Anal region	1,585	19.4
Palm of the hand	867	10.6
Oral region	806	9.9
Sole of the foot	404	4.9
Other sites	430	5.3

Approximately 28% (2,246) of cases reported being immunosuppressed, and almost half (n = 4,034; 49.4%) reported having no immunosuppression. We found that 34.6% (2,825) of confirmed and probable cases reported living with HIV and 10.5% (857) reported having an active STI, with 5.0% (412) reporting having syphilis and 2.6% (214) genital herpes (Table 4). 381 cases were hospitalized, of which 2.3% (191) were for clinical reasons, 0.6% (49) for isolation, and 1.7% (141) for unknown reasons; 18.1% (1,480) of the notifications had no information on the need for hospitalization. Of the 381 hospitalized cases, 16 were admitted to an ICU. 38% (3,109) were cured, 3.9% (319) were still being followed up in the state of São Paulo (under treatment), six cases died of other causes and 4,271 (58%) did not have information on case progression filled in on the system. Three (0.04%) deaths due to MPX were notified in the period, all of them were male, between 30 and 39 years old, living with HIV, immunosuppressed, and had been admitted to an ICU.

**Table 4:** Number and proportion of confirmed and probable monkeypox cases (n = 8,167), according to self-reported immunosuppression, living with HIV^a^ and having an active STI^b^, Brazil, June 7, 2022 (EW 23) - October 1, 2022 (EW 39)

Characteristics	n	%
Total	8,167	100.0
**Immunosuppressed**		
Yes, because of a disease	2,176	26.6
Yes, because of medication	55	0.7
Yes, cause unknown.	15	0.2
Not immunosuppressed	4,034	49.4
Not informed	1,887	23.1
**Living with HIV^a^**		
Yes	2,825	34.6
No	3,175	38.9
Not informed	2,167	26.5
**Has an active STI^b^**		
Syphilis	412	5.0
Genital herpes	214	2.6
Chlamydia	41	0.5
Gonorrhea	38	0.5
Genital warts	37	0.5
Other	115	1.4
Does nor have an active STI^b^	2,871	35.2
Not informed	4,439	54.3

a) HIV: Human immunodeficiency virus; STI: Sexually transmitted infection.

## Discussion

The study described the profile of notified confirmed and probable monkeypox cases in Brazil. We found that the outbreak in the country occurred more frequently among male and young individuals. We found a high proportion of male individuals who reported being homosexual and, according to reported sexual behavior, others who were MSM. Although MPX is not a sexually transmitted infection, an infected individual can spread the disease through prolonged and intimate contact during sexual intercourse. The high number of cases among gay men and other MSM has led the WHO to issue recommendations exclusively for this population. Participation in events where intimate contact occurred has been reported as the main mode of transmission in the recent outbreak in other countries,^1^ ^,^ ^11^ ^-^ ^15^ and it is likely that this has also been the main form of dissemination in Brazil.

This study also found an uneven distribution of cases, most of them in the Southeast, this being the region where the largest urban centers are located and where MPX entered the country, brought by international travelers. Even with the spread of cases seen in all the federative units, which presented a rising curve, followed by stabilization, an overall falling trend has been found, which corroborates the current trends of the outbreak seen globally.^16^ ^-^ ^19^

Another relevant aspect found was that most cases had mild symptoms and low hospitalization rates. In addition, three deaths were reported in Brazil, all of immunosuppressed individuals.^20^ ^,^ ^21^ However, by November 4, 2022, the Ministry of Health had recorded a further seven deaths of immunosuppressed individuals with comorbidities.^22^ Data published by the WHO also reported that most of the confirmed and probable cases worldwide were mild, with no need for hospitalization (91.6%); and when hospitalization did occur, it was for clinical reasons or for isolation (8.4%); only 0.1% of the cases led to hospitalization in ICUs. Of the total number of cases reported worldwide, 29 deaths corresponding to immunosuppressed individuals had occurred by the time this study was finished, distributed between the world’s five major regions.^19^

Most of the symptoms observed were mild, with fever, adenomegaly, headache and rash being the most frequent, while anogenital sites were the most reported (67.3%). A study conducted on 181 individuals with MPX in Spain found that all those investigated had skin lesions, 78% had lesions in the anogenital region and 43% in the oral and perioral cavity. A total of 85% had lymphadenopathy.^23^ In another study of 197 cases in England, all had mucocutaneous lesions, while fever (61.9%), lymphadenopathy (57.9%) and myalgia (31.5%) were among the most common systemic symptoms.^24^ The presence of skin rash is among the signs and symptoms most frequently recorded in the data released by the WHO on confirmed cases of MPX.^19^ These data demonstrate the high frequency of skin lesions in the cases described during the present MPX outbreak, and are an important sign for its identification.

The limitations of this study include the use of secondary data, with low completeness in the case of some of the variables, which hindered the analysis of factors of interest. Given that it is a new disease, there were also difficulties with diagnostic capacity, since laboratory diagnosis via molecular detection of the virus through real-time polymerase chain reaction (qPCR) is only performed in only eight reference laboratories in Brazil, which may have caused case underreporting. On the other hand, the data presented are of national scope and are representative of the country’s regions, and are therefore important for preparing surveillance and control actions in response to the MPX epidemic in Brazil.

Declining population immunity coupled with the discontinuation of smallpox vaccination led to the resurgence of MPX. The emergence of cases outside Africa highlighted the imminent risk of the disease spreading geographically, and thus health surveillance and case detection stand out as essential tools for understanding the changing epidemiology of MPX.^4^ The way emerging and re-emerging infectious outbreaks are handled demonstrates global negligence, especially in outbreaks of diseases and illnesses that affect Africa and underdeveloped countries. This neglect, in fact, reflects failure to invest in health strategies to find solutions for similar situations already witnessed, such as the Ebola outbreak and the covid-19 pandemic. The cut in research budgets raises the alarm about the risk of reduced resources and investment and, as a consequence, the possibility of new outbreaks, which are now on the rise.^25^

Equally important and worthy of emphasis is the way sexual orientation is communicated and correlated with the MPX virus, since this fact can generate stigmas, having the potential to delay diagnosis and keep more vulnerable groups away from access to health care.^15^

Health surveillance actions need to be strengthened, with identification of suspected and confirmed cases and active tracing of contacts, in order to contain and control infection. The duly substantiated data and guidelines described in this article provide information that, when added to the analysis of the national and international epidemiological scenarios, can generate evidence capable of informing the development of public health policies. We conclude that new studies are needed to better understand the process of how this disease is progressing in Brazil.
